# The *BRCA1*ness signature is associated significantly with response to PARP inhibitor treatment versus control in the I-SPY 2 randomized neoadjuvant setting

**DOI:** 10.1186/s13058-017-0861-2

**Published:** 2017-08-25

**Authors:** Tesa M. Severson, Denise M. Wolf, Christina Yau, Justine Peeters, Diederik Wehkam, Philip C. Schouten, Suet-Feung Chin, Ian J. Majewski, Magali Michaut, Astrid Bosma, Bernard Pereira, Tycho Bismeijer, Lodewyk Wessels, Carlos Caldas, René Bernards, Iris M. Simon, Annuska M. Glas, Sabine Linn, Laura van ‘t Veer

**Affiliations:** 1 Division of Molecular Pathology, Netherlands Cancer Institute, Amsterdam, The Netherlands; 2 University of California, San Francisco, CA USA; 3 Agendia NV, Amsterdam, The Netherlands; 4 CRUK Cambridge Institute, Cambridge, UK; 5 Division of Molecular Carcinogenesis, Netherlands Cancer Institute, Amsterdam, The Netherlands; 6 Current address: Division of Cancer and Haematology, Walter and Eliza Hall Institute of Medical Research, Melbourne, Australia; 7 Faculty of EEMCS, Delft University of Technology, Delft, The Netherlands; 8 Division Medical Oncology, Netherlands Cancer Institute, Amsterdam, The Netherlands; 9 Department of Pathology, University Medical Center Utrecht, Utrecht, The Netherlands

**Keywords:** Breast cancer, Neoadjuvant, *BRCA*ness, PARP inhibition, Triple-negative breast cancer

## Abstract

**Background:**

Patients with *BRCA1*-like tumors correlate with improved response to DNA double-strand break-inducing therapy. A gene expression-based classifier was developed to distinguish between *BRCA1*-like and non-*BRCA1*-like tumors. We hypothesized that these tumors may also be more sensitive to PARP inhibitors than standard treatments.

**Methods:**

A diagnostic gene expression signature (*BRCA1*ness) was developed using a centroid model with 128 triple-negative breast cancer samples from the EU FP7 RATHER project. This *BRCA1*ness signature was then tested in HER2-negative patients (*n* = 116) from the I-SPY 2 TRIAL who received an oral PARP inhibitor veliparib in combination with carboplatin (V-C), or standard chemotherapy alone. We assessed the association between *BRCA1*ness and pathologic complete response in the V-C and control arms alone using Fisher’s exact test, and the relative performance between arms (biomarker × treatment interaction, likelihood ratio *p* < 0.05) using a logistic model and adjusting for hormone receptor status (HR).

**Results:**

We developed a gene expression signature to identify *BRCA1*-like status. In the I-SPY 2 neoadjuvant setting the *BRCA1*ness signature associated significantly with response to V-C (*p* = 0.03), but not in the control arm (*p* = 0.45). We identified a significant interaction between *BRCA1*ness and V-C (*p* = 0.023) after correcting for HR.

**Conclusions:**

A genomic-based *BRCA1*-like signature was successfully translated to an expression-based signature (*BRC1A*ness). In the I-SPY 2 neoadjuvant setting, we determined that the *BRCA1*ness signature is capable of predicting benefit of V-C added to standard chemotherapy compared to standard chemotherapy alone.

**Trial registration:**

I-SPY 2 TRIAL beginning December 31, 2009: Neoadjuvant and Personalized Adaptive Novel Agents to Treat Breast Cancer (I-SPY 2), NCT01042379.

**Electronic supplementary material:**

The online version of this article (doi:10.1186/s13058-017-0861-2) contains supplementary material, which is available to authorized users.

## Background

Histological subtype information in breast cancer is clinically relevant for treatment purposes but insufficient to describe all tumor heterogeneity [1]. The basal-like molecular subtype, which typically expresses cytokeratins 5/6 and EGFR, frequently overlaps with the histological subtype of triple-negative (TN) breast cancer [2, 3]. TN breast cancer is described by the absence of ERα/PR and HER2 expression and poor overall prognosis [4, 5]. Because of the lack of available targeted therapies for this subtype, the clinical impact of target discovery for patients with TN breast cancer is potentially significant.

Hereditary germline *BRCA1* mutations are found in around 12% of all TN breast cancers [6–8]. BRCA1 plays a critical role in error-free DNA double-strand break repair via homologous recombination, and deficiency can result in genomic instability [9, 10]. Differential gene expression patterns in *BRCA1* mutant tumors versus nonmutant tumors have been identified previously [11–14]. Because of the relative rarity of *BRCA1* mutation in the general breast cancer population [15], however, these studies are often underpowered, making clinical impact for mutation carriers limited. Furthermore, the capacity of these signatures to predict response to targeted treatments such as PARP inhibitors has not been thoroughly explored in the randomized clinical trial setting.

BRCA1-mutated/promoter-methylated TN tumors with a specific pattern of copy number alterations are termed *BRCA1*-like [16–20]. ‘*BRCA*ness’ describes tumors with molecular features of BRCA1-mutated tumors [21, 22]. Interestingly, the whole group of *BRCA1*-like tumors responds well to DNA double-strand break-inducing agents and intensifying chemotherapy regardless of their *BRCA1* mutation/promoter methylation status [16, 23, 24]. These findings suggest that a relatively large portion of TN breast cancers may be susceptible to targeted therapies such as PARP inhibitors. The efforts of many groups have resulted in various classifiers for *BRCA*ness, typically based on mutation [13, 14, 25] or homologous recombination repair deficiency (HRD) markers [26] and using gene expression data as an input. Recent work has found that an assay designed to detect *BRCA*ness using HRD as a biomarker failed to predict for carboplatin response [27], illustrating the challenges of generating a signature with the capacity to predict treatment effect [28].

Molecular subgroups within the TN subtype have differential benefit from therapies [29–31]. In addition, previous work in TN tumors has determined that differentially expressed genes between *BRCA1*-like and non-*BRCA1*-like tumors center around DNA repair [29, 32, 33] and may lead to new information for clinical therapeutic decisions. A test based on gene expression levels may also lead to insight into the mechanisms which result in tumors with *BRCA1*-like features. We developed a 77-gene signature to identify samples with a *BRCA1*-like gene expression pattern we term *BRCA1*ness with a sensitivity and specificity of 96.7% and 73.1%, respectively. We explored this signature’s ability to predict response to the PARP inhibitor veliparib in combination with carboplatin (V-C) in the I-SPY 2 TRIAL, a phase 2, multicenter, adaptively randomized trial designed to screen multiple experimental regimens in combination with standard neoadjuvant chemotherapy for breast cancer, where V-C graduated in the TN signature [34–36]. Investigation of the *BRCA1*ness signature was part of a further evaluation of additional biomarkers in this setting. In this study, we aimed to answer the clinical question in the I-SPY 2 external validation set of whether to treat with PARP inhibition based on the well-studied mechanism of HRD (identified by our biomarker *BRCA1*ness).

## Methods

### Discovery set patient characterization and microarray data generation

The collaborative European Union-funded effort FP7 RATHER project (*Ra*tional *Ther*apy for Breast Cancer) aims to integrate gene expression profiling, copy number variation, kinome variation and kinase activation status in an effort to identify new targets for therapy of difficult-to-treat breast cancer subtypes, including TN breast cancer (www.ratherproject.com). The RATHER project retrospectively identified 128 TN breast cancer patients with long-term follow-up in total: 70 from Netherlands Cancer Institute (NKI), Amsterdam, the Netherlands and 58 from Addenbrooke’s Hospital, Cambridge, UK.

The primary inclusion criterion for the RATHER cohort was availability of sufficient isolated frozen RNA tissue in the tissue bank and diagnostic information indicative of TN breast cancer. We enriched for frozen tumors with 30% or greater tumor content (2 x 8-μm serial sections, hematoxylin and eosin stained). The local medical ethics authorities of both centers approved the collection protocols. Sectioning of tumor tissue and RNA isolation were performed as described previously [37].

Samples with RIN value > 5 according to 2100 Bioanalyzer (Agilent Technologies) assessment were selected for further analysis. Gene expression data were generated as described previously [37]. Briefly, feature signal intensities were processed and extracted using the ‘limma’ R package with background subtraction using an offset of 10 and log_2_ transformed data. Probe intensities were quantile normalized with in-house R scripts and missing values (including probes with signal intensities < 1 after preprocessing) were imputed by the 10 nearest-neighbor method. A biobank batch effect was adjusted using ComBat [38]. Genes with multiple probes were summarized by the first principal component of a correlating subset.

### *BRCA1*-like classification

The multiplex ligation-dependent probe amplification (MLPA) method was used to generate copy number profiles for the determination of the *BRCA1*-like status of the tumors. The assay was performed, fragments analyzed and data normalized according to the manufacturer’s protocol (MRC-Holland). Class prediction (*BRCA1*-like/non-*BRCA1*-like) was carried out on the normalized data according to published instructions [16].

### Gene signature development

Signature development was performed using Partek Genomics Suite (partek.com) (categorical signature) and Matlab (https://mathworks.com/) (translation to continuous score) on 128 samples. Top variable genes (variance >1 across all samples) were used for the model input (*N* = 2049). The classification model of diagonal linear discriminant analysis (DLDA) with equal prior probabilities was run to select the signature genes. Groups of genes ranked by their significance in a univariate ANOVA examining the *BRCA1*-like/non-*BRCA1*-like MLPA status were tested (groups from 1 to 100, in increments of 1). Single-level leave-one-out cross validation (LOOCV) with the maximum number of partitions was used to internally validate and calculate the performance of the model.

The significant number of genes in the model (*n* = 77, Additional file 1: Table S1) was selected based on the ROC area under the curve (AUC as specified by Partek). After the model is run, each sample is allocated a posterior probability for each class (*BRCA1*-like and non-*BRCA1*-like) and the sample is assigned to the class with the highest posterior probability (*BRCA1*ness). This categorical signature was then transferred to the diagnostic setting to better comply with quality and regulatory requirements using a nearest centroid model; a robust method with both reasonable and favorable characteristics for many measurements on a modest amount of patients [39]. Briefly, raw full genome data were normalized and class centroids were calculated (median per gene) for each of the 77 genes for each class (*BRCA1*ness/non-*BRCA1*ness) using the discovery set. These calculated centroids were used as the template for *BRCA1*ness/non-*BRCA1*ness. Pearson correlations of each new sample with the *BRCA1*ness/non-*BRCA1*ness templates were calculated (Additional file 1: Table S1) and combined into a single continuous score by subtracting the correlation to the non-*BRC1A*ness template from the correlation to the *BRC1A*ness template. In order for a sample to be classified as *BRCA1*ness, a threshold was established with a high sensitivity while preserving specificity close to 0.75. The classification threshold was set at –0.3; that is, a sample with a *BRCA1*ness score > –0.3 was classified as *BRCA1*ness and a sample with a score < –0.3 was classified as non-*BRCA1*ness.

### I-SPY 2 TRIAL

**Fig. 1:**
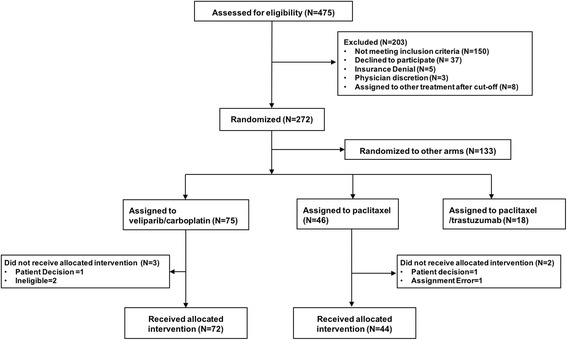
CONSORT diagram. CONSORT diagram indicating how patients were randomized for the I-SPY 2 TRIAL

The I-SPY 2 TRIAL is a standing multicenter, phase 2 platform trial to screen experimental regimens in combination with standard chemotherapy in the neoadjuvant treatment of breast cancer. Patients are adaptively randomized into one of four experimental arms or a control arm (Fig. 1) [35]. In this portion of the I-SPY 2 TRIAL, eligible patients received weekly paclitaxel at 80 mg/m^2^ (T) i.v. for 12 doses alone (control), or in combination with an experimental regimen. Patients randomized to V-C received 50 mg of veliparib by mouth twice daily for 12 weeks and carboplatin at AUC 6 on weeks 1, 4, 7 and 10 concurrent with weekly paclitaxel. Following paclitaxel ± V-C, all patients received doxorubicin 60 mg/m^2^ and cyclophosphamide 600 mg/m^2^ (AC) i.v. every 2–3 weeks for four doses with myeloid growth factor support as appropriate per protocol followed by surgery that included axillary node sampling. The V-C arm was open to HER2-negative patients; and was graduated in the TN group. The *BRCA1*ness signature was one of the qualifying dichotomous biomarkers assessed as a predictor of response to V-C relative to standard chemotherapy.

To assess the *BRCA1*ness signature in this validation set as a specific biomarker of V-C response, gene expression data from 116 HER2-negative patients (V-C, *n* = 72 and concurrent controls, *n* = 44) were analyzed. A Customized Agilent 44 K array (Agendia) was used to evaluate the 77-gene signature *BRCA1*ness classification. The association between *BRCA1*ness classification and response in the V-C and control arms alone was assessed using Fisher’s exact test, and the relative performance between arms (biomarker × treatment interaction, likelihood ratio test) using a logistic model. We included adjustment for hormone receptor status (HR/TN) and tumor size in our model. Our sample size is small, and thus statistical calculations (*p* values) are descriptive rather than inferential. This analysis does not adjust for multiplicities of other biomarkers evaluated in the trial but outside this study.

## Results

### Signature development

We developed a *BRCA1*ness signature using whole genome gene expression data. The signature has been developed on fresh frozen (FF) breast tumors that were categorized as either *BRCA1*-like or non-*BRCA1*-like using a DNA copy number MLPA-based classifier [16], and endeavors to predict *BRCA1*-like tumors with a high sensitivity/specificity rate.

Forty-eight percent of the tumors (61/128) in the discovery cohort were classified as *BRCA1*-like and the remainder was assigned to the non-*BRCA1*-like class. We employed the gene expression profiles of the tumors, DLDA and the labels assigned by the MLPA-based classifier to train a classifier that distinguishes between the two classes. Using the ROC area under the curve model (AUC) as the performance criterion we identified a 77-gene signature that resulted in the highest performance (Table 1).

**Table 1:** Sensitivity and specificity for detecting *BRCA1*-like status samples using *BRCA1*ness

	MLPA *BRCA1*-like	MLPA non-*BRCA1*-like
*BRCA1*ness positive	59	18
*BRCA1*ness negative	2	49
	Sensitivity	Specificity
	96.7%	73.1%

*MLPA* multiplex ligation-dependent probe amplification

**Fig. 2:**
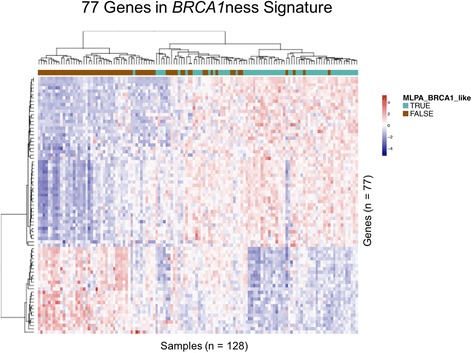
Unsupervised hierarchical clustering of 77 genes in the 128 discovery set samples. The 77 genes were derived from a supervised analysis to identify those genes most informative in distinguishing *BRCA1*-like from non-*BRCA1*-like TN breast cancers [33]. Scaled expression value denoted as *Z* score (*red–blue* scale: *red* indicates high expression and *blue* indicates low expression). Information bar indicates MLPA *BRCA1*-like status: true (*green*) or false (*brown*). *MLPA* multiplex ligation-dependent probe amplification (Color figure online)

**Fig. 3:**
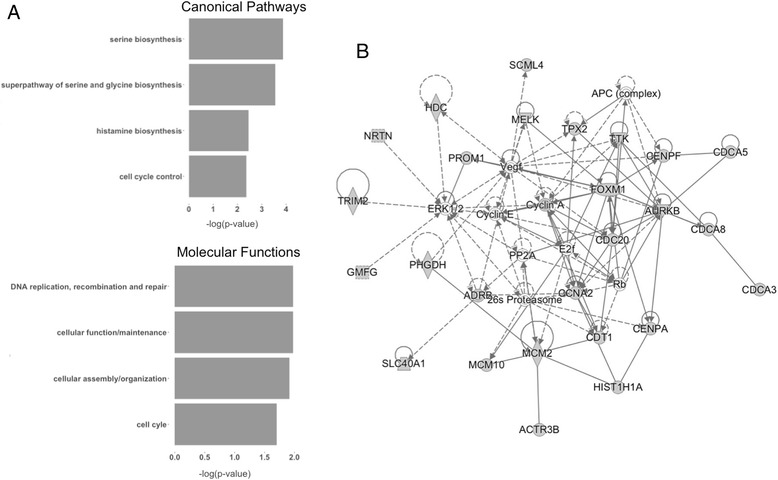
The 77-gene signature network analysis. **a** Significant canonical pathways (*top*) and molecular functions (*bottom*). Negative log *p* value is on the *x* axis. **b** Network analysis of the 77 genes in the *BRCA1*ness signature. *Grey shading* indicates genes found in signature, *solid lines* show direct relationships between proteins and *dashed lines* show indirect relationships

Unsupervised hierarchical clustering of the genes in the 77-gene signature indicates separation between the classes (Fig. 2). We transferred the signature to a diagnostic setting using a nearest centroid-based algorithm. The sensitivity and specificity for detecting *BRCA1*-like status as defined by MLPA were 96.7% and 73.1%, respectively. Using Ingenuity Pathway Analysis (Qiagen) to identify key biological processes associated with the 77 genes in the *BRCA1*ness 77-gene signature, we found cellular assembly and control and DNA replication, recombination and repair to be among the top associated pathways and functions (Fig. 3a). In addition, we observed serine and glycine biosynthesis to be associated with the 77-gene signature genes, indicating that these genes may be responsible for reprogramming of metabolic processes, which can lead to tumor progression (Fig. 3a). Supporting the pathway and molecular function results, network analysis revealed a network centered upon cell the cycle control regulator cyclin A (Fig. 3b).

### I-SPY 2 TRIAL

**Fig. 4:**
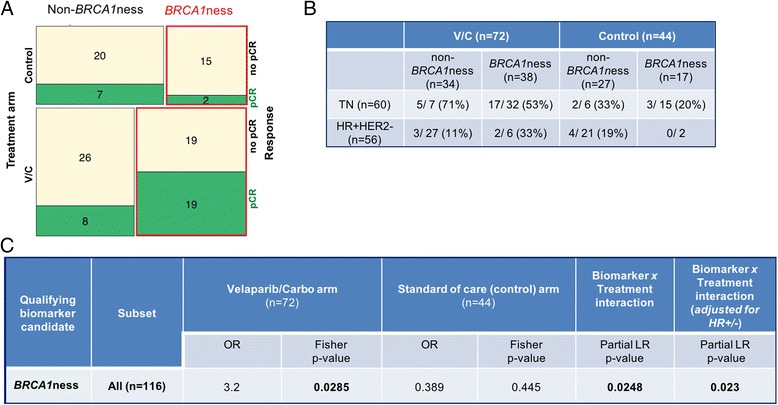
I-SPY 2 TRIAL. **a** Mosaic plot depicting the number of patients with pathological complete response (*pCR*) in each treatment group and signature group. *Top row* indicates patients in the trial enrolled in the control arm and *bottom row* indicates patients in the V-C arm. Number of patients with pCR is shown in *green* and number of patients without pCR is shown in *tan. Black outlined boxes* indicate the patients with a non-*BRCA1*ness status (*left*), *red outlined boxes* depict those with *BRCA1*ness status (*right*). **b** Histological subtype of the patients in the trial divided by treatment arm (V-C) and control arm and pCR rate per group. **c** Odds ratio (*OR*) and likelihood ratio test (*LR*) for treatment and control arms of the trial and the biomarker x treatment interaction test. *HER2* human epidermal growth factor receptor 2, *HR* hormone receptor status, *TN* triple-negative, *V/C* veliparib-carboplatin (Color figure online)

The *BRCA1*ness signature was applied to 116 HER2-negative patients (V-C, *n* = 72 and concurrent controls, *n* = 44). Fifty-five patients were classified as *BRCA1*ness. Fourteen percent of these patients were hormone receptor-positive (ERα/PR) and HER2-negative. The distribution of pathological complete response (pCR) rates among *BRCA1*ness and non-*BRCA1*ness groups is shown in Fig. 4a [36] and Table 2. Association between the *BRCA1*ness classification and patient response was seen in the V-C arm (OR = 3.2, *p* = 0.03) but not in the control arm (OR = 0.39, *p* = 0.45) (Fig. 4b). A significant biomarker × treatment interaction (*p* = 0.025) was also observed. Although there is enrichment for TN samples in the *BRCA1*ness group in univariate analysis (Table 2), this interaction remains significant upon adjusting for HR (*p* = 0.023) (Fig. 4c).

**Table 2:** Patient characteristics

Variable	Non-*BRCA1*ness	*BRCA1n*ess	*p* value
	(*N* = 61)	(*N* = 55)	
Treatment			
Control	27 (44.3)	17 (30.9)	0.198^a^
Veliparib/carboplatin	34 (55.7)	38 (69.1	
Hormone receptor status			
HR+	48 (78.7)	8 (14.5)	<0.05^a^
TN	13 (21.3)	47 (85.5)	
Tumor size (cm)			
0–1	0 (0)	0 (0)	0.496^a^
>1–2	0 (0)	1 (1.8)	
>2–5	40 (65.6)	31 (56.4)	
>5	19 (31.4)	22 (40.0)	

Data presented as number (%)

*HR* hormone receptor status, *TN* triple-negative

^a^Pearson’s chi-squared test

In addition we found that the interaction also remains significant when adjusting for tumor size and HR (*p* = 0.038). We use the likelihood ratio test to formally demonstrate that the logistic regression with the addition of the HR (and tumor size) terms does not provide a better fit to the data. When the hormone receptor-positive *BRCA1*ness classified patients are added to the graduating TN subset, the OR associated with V-C is 4.03. This is comparable to that of the TN alone (OR = 4.04), while increasing the prevalence of ‘biomarker-positive’ predicted V-C sensitive patients by 8%.

## Discussion

At around 15% of all breast cancers, the TN breast cancer subtype impacts a significant proportion of women [7, 40]. TN breast cancer tends to be aggressive independent of other known prognostic factors [5, 41]. Current guidelines indicate that standard therapy for TN breast cancer is chemotherapy [2]. Unfortunately, these tumors typically metastasize early despite therapy [5, 41]. This poor response to treatment may be due to the fact that the TN subtype itself is made of molecular subgroups. Conversely, molecular data from these subgroups may indicate a targeted therapy, which is likely to benefit patients.

Because of results in preclinical models, *BRCA1* mutation carriers of multiple tumor types have been enrolled in clinical trials with PARP inhibitors [27, 32, 42–44]. Currently, there are no other predictive biomarkers for PARP inhibitors other than the germline BRCA1 mutation status, and the issue with that biomarker is that it only captures a small subgroup of all patients that may benefit from carboplatin/veliparib [8]. Previous work has shown that genomic instability patterns are related to *BRCA1* mutation/methylation and that these patterns can be used to classify tumors are *BRCA1*-like or non-BRCA1-like [16–20, 24]. These *BRCA1*-like tumors make up a larger group than *BRCA1* mutant or methylated alone, and importantly they respond well to DNA double-strand break-inducing chemotherapies [17, 23, 24]. We have developed a gene expression signature that is capable of identifying *BRCA1*-like samples with a high sensitivity/specificity rate (*BRCA1*ness).

Pathway analysis reveals that the genes in this signature are associated closely with cell cycle and cancer networks. We also observed a significant association with serine and glycine biosynthesis pathways with the genes of the signature. This is of particular interest because it has been recently shown that aerobic glycolysis signaling can promote tumor growth in breast cancer cell lines that are TN [45], indicating that the poor outcome for patients with a *BRCA1*ness tumor may be partially explained in this manner. Serine biosynthesis has been identified to be essential to tumorigenesis in estrogen receptor-negative breast cancer cell lines [46]. We identified serine biosynthesis to be related specifically to the genes found in the *BRCA1*ness gene expression signature, suggesting that tumors having *BRCA1*-like features may have particular vulnerabilities to drugs that interfere with serine biosynthesis. It would be interesting to test whether high expression of genes involved in serine and glycine biosynthesis can confer sensitivity to drugs which interfere with this biosynthesis in breast cancer cell lines. In addition, we found that the signature is capable of predicting response to the PARP inhibitor veliparib in combination with carboplatin compared with a control treatment regimen.

## Conclusion

The sample size in the I-SPY 2 TRIAL is small, but our prespecified analysis suggests that the *BRCA1*ness signature shows promise for predicting response to V-C combination therapy relative to control. We focused on the experimental arm of the study that contains DNA-damaging agents because the *BRCA1*ness test is meant to identify patients that may derive substantial benefit from these agents. We observed a proportion of patients who were hormone receptor-positive that benefited from the V-C treatment. It is unlikely in a regular clinical setting that hormone receptor-positive patients would be tested for *BRCA1*ness, but our data indicate that these patients could derive benefit from specific tailored treatments like PARP inhibitors and/or platinum agents. Concurrently reported results studying carboplatin in TN breast cancer have indicated it may be difficult to translate the pCR rate to longer benefit such as recurrence-free survival (RFS) [47–50]. It should be noted that, for this trial, we used a surrogate endpoint (pCR) for RFS and longer follow-up is required to investigate the *BRCA1*ness classifier in relation to long-term benefit. In the event that downsizing of the tumor is required to facilitate conversion from mastectomy to breast-conserving therapy, this classifier may already have value. If verified in a larger trial, this signature may contribute to the selection criteria of PARP inhibitor trials in the future.

## Additional files

Additional file 1: *BRCA1*ness signature genes. (XLSX 40 kb)

Additional file 2: I-SPY 2 Trial participating sites and institutional review boards (IRB). (XLSX 48 kb)

## Abbreviations

ANOVA: Analysis of variance
AUC: Area under the curve
DLDA: Diagonal linear discriminant analysis
EGFR: Epidermal growth factor
ERα: Estrogen receptor alpha
EU FP7: European Union 7th Framework Programme
FF: Fresh frozen
HER2: Human epidermal growth factor receptor 2
HR: Hormone receptor status
HRD: Homologous repair deficiency
I-SPY 2 TRIAL: Neoadjuvant and Personalized Adaptive Novel Agents to Treat Breast Cancer
LOOCV: Leave-one-out cross validation
MLPA: Multiplex ligation-dependent probe amplification
NKI: Netherlands Cancer Institute
OR: Odds ratio
PARP: Poly (ADP-ribose) polymerase
pCR: Pathological complete response
PR: Progesterone receptor
RATHER: Rational Therapy for Breast Cancer
RIN: RNA integrity number
ROC: Receiver-operating characteristic
TN: Triple-negative
V-C: Veliparib–carboplatin

## References

[1] Curtis C, Shah SP, Chin S-F, Turashvili G, Rueda OM, Dunning MJ (2012). The genomic and transcriptomic architecture of 2,000 breast tumours reveals novel subgroups. Nature.

[2] Linn SC, van ‘t Veer LJ (2009). Clinical relevance of the triple-negative breast cancer concept: genetic basis and clinical utility of the concept. Eur J Cancer Oxf Engl 1990.

[3] Prat A, Adamo B, Cheang MCU, Anders CK, Carey LA, Perou CM (2013). Molecular characterization of basal-like and non-basal-like triple-negative breast cancer. Oncologist.

[4] Nielsen TO, Hsu FD, Jensen K, Cheang M, Karaca G, Hu Z (2004). Immunohistochemical and clinical characterization of the basal-like subtype of invasive breast carcinoma. Clin Cancer Res Off J Am Assoc Cancer Res.

[5] Blows FM, Driver KE, Schmidt MK, Broeks A, van Leeuwen FE, Wesseling J (2010). Subtyping of breast cancer by immunohistochemistry to investigate a relationship between subtype and short and long term survival: a collaborative analysis of data for 10,159 cases from 12 studies. PLoS Med.

[6] Collins LC, Martyniak A, Kandel MJ, Stadler ZK, Masciari S, Miron A (2009). Basal cytokeratin and epidermal growth factor receptor expression are not predictive of BRCA1 mutation status in women with triple-negative breast cancers. Am J Surg Pathol.

[7] Foulkes WD, Stefansson IM, Chappuis PO, Bégin LR, Goffin JR, Wong N (2003). Germline BRCA1 mutations and a basal epithelial phenotype in breast cancer. J Natl Cancer Inst.

[8] Hartman A-R, Kaldate RR, Sailer LM, Painter L, Grier CE, Endsley RR (2012). Prevalence of BRCA mutations in an unselected population of triple-negative breast cancer. Cancer.

[9] Bouwman P, Jonkers J (2012). The effects of deregulated DNA damage signalling on cancer chemotherapy response and resistance. Nat Rev Cancer.

[10] Moynahan ME, Chiu JW, Koller BH, Jasin M (1999). Brca1 controls homology-directed DNA repair. Mol Cell.

[11] Hedenfalk I, Duggan D, Chen Y, Radmacher M, Bittner M, Simon R (2001). Gene-expression profiles in hereditary breast cancer. N Engl J Med.

[12] Jönsson G, Naylor TL, Vallon-Christersson J, Staaf J, Huang J, Ward MR (2005). Distinct genomic profiles in hereditary breast tumors identified by array-based comparative genomic hybridization. Cancer Res.

[13] van’t Veer LJ, Dai H, van de Vijver MJ, He YD, Hart AAM, Mao M (2002). Gene expression profiling predicts clinical outcome of breast cancer. Nature.

[14] Larsen MJ, Kruse TA, Tan Q, Lænkholm A-V, Bak M, Lykkesfeldt AE (2013). Classifications within molecular subtypes enables identification of BRCA1/BRCA2 mutation carriers by RNA tumor profiling. PLoS One.

[15] Pereira B, Chin S-F, Rueda OM, Vollan H-KM, Provenzano E, Bardwell HA (2016). The somatic mutation profiles of 2,433 breast cancers refines their genomic and transcriptomic landscapes. Nat Commun..

[16] Lips EH, Laddach N, Savola SP, Vollebergh MA, Oonk AMM, Imholz ALT (2011). Quantitative copy number analysis by Multiplex Ligation-dependent Probe Amplification (MLPA) of BRCA1-associated breast cancer regions identifies BRCAness. Breast Cancer Res.

[17] Lips EH, Mulder L, Hannemann J, Laddach N, Vrancken Peeters MTFD, van de Vijver MJ (2011). Indicators of homologous recombination deficiency in breast cancer and association with response to neoadjuvant chemotherapy. Ann Oncol.

[18] Vollebergh MA, Jonkers J, Linn SC (2012). Genomic instability in breast and ovarian cancers: translation into clinical predictive biomarkers. Cell Mol Life Sci.

[19] Schouten PC, van Dyk E, Braaf LM, Mulder L, Lips EH, de Ronde JJ (2013). Platform comparisons for identification of breast cancers with a BRCA-like copy number profile. Breast Cancer Res Treat.

[20] Wessels LFA, van Welsem T, Hart AAM, van’t Veer LJ, Reinders MJT, Nederlof PM (2002). Molecular classification of breast carcinomas by comparative genomic hybridization: a specific somatic genetic profile for BRCA1 tumors. Cancer Res.

[21] Lord CJ, Ashworth A (2016). BRCAness revisited. Nat Rev Cancer.

[22] Turner N, Tutt A, Ashworth A (2004). Hallmarks of “BRCAness” in sporadic cancers. Nat Rev Cancer.

[23] Schouten PC, Marmé F, Aulmann S, Sinn H-P, van Essen HF, Ylstra B (2015). Breast cancers with a BRCA1-like DNA copy number profile recur less often than expected after high-dose alkylating chemotherapy. Clin Cancer Res.

[24] Vollebergh MA, Lips EH, Nederlof PM, Wessels LFA, Schmidt MK, van Beers EH (2011). An aCGH classifier derived from BRCA1-mutated breast cancer and benefit of high-dose platinum-based chemotherapy in HER2-negative breast cancer patients. Ann Oncol.

[25] Konstantinopoulos PA, Spentzos D, Karlan BY, Taniguchi T, Fountzilas E, Francoeur N (2010). Gene expression profile of BRCAness that correlates with responsiveness to chemotherapy and with outcome in patients with epithelial ovarian cancer. J Clin Oncol.

[26] Peng G, Chun-Jen Lin C, Mo W, Dai H, Park Y-Y, Kim SM (2014). Genome-wide transcriptome profiling of homologous recombination DNA repair. Nat Commun..

[27] Tutt A, Ellis P, Kilburn L, Gilett C, Pinder S, Abraham J, et al. [S3-01] The TNT trial: a randomized phase III trial of carboplatin (C) compared with docetaxel (D) for patients with metastatic or recurrent locally advanced triple negative or BRCA1/2 breast cancer (CRUK/07/012). Cancer Res. 2015;75(9 Supplement):S3-01-S3-01.

[28] Schouten PC, Linn SC (2015). Challenges in the use of DNA repair deficiency as a biomarker in breast cancer. J Clin Oncol.

[29] Lehmann BD, Bauer JA, Chen X, Sanders ME, Chakravarthy AB, Shyr Y (2011). Identification of human triple-negative breast cancer subtypes and preclinical models for selection of targeted therapies. J Clin Invest.

[30] Masuda H, Baggerly KA, Wang Y, Zhang Y, Gonzalez-Angulo AM, Meric-Bernstam F (2013). Differential response to neoadjuvant chemotherapy among 7 triple-negative breast cancer molecular subtypes. Clin Cancer Res.

[31] Yu K-D, Zhu R, Zhan M, Rodriguez AA, Yang W, Wong S (2013). Identification of prognosis-relevant subgroups in patients with chemoresistant triple-negative breast cancer. Clin Cancer Res.

[32] Farmer H, McCabe N, Lord CJ, Tutt ANJ, Johnson DA, Richardson TB (2005). Targeting the DNA repair defect in BRCA mutant cells as a therapeutic strategy. Nature.

[33] Severson TM, Peeters J, Majewski I, Michaut M, Bosma A, Schouten PC, et al. BRCA1-like signature in triple negative breast cancer: molecular and clinical characterization reveals subgroups with therapeutic potential. Mol Oncol. 2015;8(7):1528–38. 10.1016/j.molonc.2015.04.011 PMC5528786 26004083

[34] Park JW, Liu MC, Yee D, Yau C, van’t Veer LJ, Symmans WF (2016). Adaptive randomization of neratinib in early breast cancer. N Engl J Med.

[35] Rugo HS, Olopade OI, DeMichele A, Yau C, van ‘t Veer LJ, Buxton MB (2016). Adaptive randomization of veliparib-carboplatin treatment in breast cancer. N Engl J Med.

[36] Wolf DM, Yau C, Sanil A, Glas A, Petricoin C, Wulfkuhle J, et al. DNA repair deficiency biomarkers and the 70-gene ultra-high risk signature as predictors of veliparib/carboplatin response in the I-SPY 2 breast cancer trial. NPJ Breast Cancer (2017). http://dx.doi.org/10.1038/s41523-017-0025-7 10.1038/s41523-017-0025-7 PMC5572474 28948212

[37] Michaut M, Chin S-F, Majewski I, Severson TM, Bismeijer T, de Koning L (2016). Integration of genomic, transcriptomic and proteomic data identifies two biologically distinct subtypes of invasive lobular breast cancer. Sci Rep..

[38] Johnson WE, Li C, Rabinovic A (2007). Adjusting batch effects in microarray expression data using empirical Bayes methods. Biostat Oxf Engl.

[39] Glas AM, Floore A, Delahaye LJMJ, Witteveen AT, Pover RCF, Bakx N (2006). Converting a breast cancer microarray signature into a high-throughput diagnostic test. BMC Genomics..

[40] Criscitiello C, Azim HA, Schouten PC, Linn SC, Sotiriou C (2012). Understanding the biology of triple-negative breast cancer. Ann Oncol.

[41] Hudis CA, Gianni L (2011). Triple-negative breast cancer: an unmet medical need. Oncologist..

[42] Fong PC, Boss DS, Yap TA, Tutt A, Wu P, Mergui-Roelvink M (2009). Inhibition of poly(ADP-ribose) polymerase in tumors from BRCA mutation carriers. N Engl J Med.

[43] Lee J-M, Ledermann JA, Kohn EC (2014). PARP Inhibitors for BRCA1/2 mutation-associated and BRCA-like malignancies. Ann Oncol.

[44] Tutt A, Robson M, Garber JE, Domchek SM, Audeh MW, Weitzel JN (2010). Oral poly(ADP-ribose) polymerase inhibitor olaparib in patients with BRCA1 or BRCA2 mutations and advanced breast cancer: a proof-of-concept trial. Lancet.

[45] Lim S-O, Li C-W, Xia W, Lee H-H, Chang S-S, Shen J (2016). EGFR signaling enhances aerobic glycolysis in triple-negative breast cancer cells to promote tumor growth and immune escape. Cancer Res.

[46] Possemato R, Marks KM, Shaul YD, Pacold ME, Kim D, Birsoy K (2011). Functional genomics reveal that the serine synthesis pathway is essential in breast cancer. Nature.

[47] von Minckwitz G, Schneeweiss A, Loibl S, Salat C, Denkert C, Rezai M (2014). Neoadjuvant carboplatin in patients with triple-negative and HER2-positive early breast cancer (GeparSixto; GBG 66): a randomised phase 2 trial. Lancet Oncol.

[48] von Minckwitz G, Loibl S, Schneeweiss A, Salat C, Rezai M, Zahm DM, et al. Early survival analysis of the randomized phase II trial investigating the addition of carboplatin to neoadjuvant therapy for triple-negative and HER2-positive early breast cancer (GeparSixto). Cancer Res. 2016;76(4 Supplement):S2-04-S2-04.

[49] Sikov WM, Berry DA, Perou CM, Singh B, Cirrincione CT, Tolaney SM (2015). Impact of the addition of carboplatin and/or bevacizumab to neoadjuvant once-per-week paclitaxel followed by dose-dense doxorubicin and cyclophosphamide on pathologic complete response rates in stage II to III triple-negative breast cancer: CALGB 40603 (Alliance). J Clin Oncol.

[50] Sikov WM, Berry DA, Perou CM, Singh B, Cirrincione CT, Tolaney SM, et al. Event-free and overall survival following neoadjuvant weekly paclitaxel and dose-dense AC +/– carboplatin and/or bevacizumab in triple-negative breast cancer: outcomes from CALGB 40603 (Alliance). Cancer Res. 2016;76(4 Supplement):S2-05-S2-05.

